# Novel Analysis of Immune Cells from Nasal Microbiopsy Demonstrates Reliable, Reproducible Data for Immune Populations, and Superior Cytokine Detection Compared to Nasal Wash

**DOI:** 10.1371/journal.pone.0169805

**Published:** 2017-01-20

**Authors:** Simon P. Jochems, Katherine Piddock, Jamie Rylance, Hugh Adler, Beatriz F. Carniel, Andrea Collins, Jenna F. Gritzfeld, Carole Hancock, Helen Hill, Jesus Reiné, Alexandra Seddon, Carla Solórzano, Syba Sunny, Ashleigh Trimble, Angela D. Wright, Seher Zaidi, Stephen B. Gordon, Daniela M. Ferreira

**Affiliations:** 1 Department of Clinical Sciences, Liverpool School of Tropical Medicine, Liverpool, United Kingdom; 2 Department of Respiratory Medicine, Aintree University Hospital NHS Trust, Liverpool, United Kingdom; 3 Department of Respiratory Medicine, Royal Liverpool University Hospital, Liverpool, United Kingdom; 4 National Institute of Health and Research Clinical Research Network, Leeds, United Kingdom; 5 Malawi Liverpool Wellcome Trust Clinical Research Programme, Blantyre, Malawi; Imperial College London, UNITED KINGDOM

## Abstract

The morbidity and mortality related to respiratory tract diseases is enormous, with hundreds of millions of individuals afflicted and four million people dying each year. Understanding the immunological processes in the mucosa that govern outcome following pathogenic encounter could lead to novel therapies. There is a need to study responses at mucosal surfaces in humans for two reasons: (i) Immunological findings in mice, or other animals, often fail to translate to humans. (ii) Compartmentalization of the immune system dictates a need to study sites where pathogens reside. In this manuscript, we describe two novel non-invasive nasal mucosal microsampling techniques and their use for measuring immunological parameters: 1) using nasal curettes to collect cells from the inferior turbinate and; 2) absorptive matrices to collect nasal lining fluid. Both techniques were well tolerated and yielded reproducible and robust data. We demonstrated differences in immune populations and activation state in nasal mucosa compared to blood as well as compared to nasopharyngeal lumen in healthy adults. We also found superior cytokine detection with absorptive matrices compared to nasal wash. These techniques are promising new tools that will facilitate studies of the immunological signatures underlying susceptibility and resistance to respiratory infections.

## Introduction

Respiratory tract disease is an important cause of morbidity and mortality worldwide [1]. In addition, lower respiratory tract infections and chronic respiratory disease, such as asthma, have both been highlighted as leading causes of disability [2, 3]. The nasal mucosa is the key niche in the pathogenesis of respiratory disease. For example, carriage of *Streptococcus pneumoniae* (the pneumococcus) in the nasopharynx has been identified as an essential step in developing both localised and systemic infection [4]. Alteration of the nasal mucosa, for example by viruses, has also been shown to increase susceptibility to pneumococcal infection [5]. Moreover, viral infection of the upper respiratory tract is associated with exacerbation of asthma and excessive inflammation in the upper respiratory tract is a risk factor for asthma [6, 7]. A greater understanding of the immunological responses to pathogens in the nasal mucosa and the pathogenesis of respiratory diseases may provide targets for new treatments or vaccinations against disease.

There is increasing evidence that mucosal immune responses vary significantly at different sites within the body [8, 9]. This compartmentalisation necessitates specific study of the nasal mucosa as it is a key component of host-pathogen interaction. Developing non-invasive techniques to study the nasal mucosa in humans offers clear advantages over animal models, which frequently lack translational applicability [10].

Currently, the most used method for collecting cells from within the nasopharynx is a nasal wash (NW) procedure [11]. The NW procedure is generally well tolerated but not suitable for all groups of patients especially those who are particularly young or unwell. In addition, luminal cell populations can vary significantly from intra-mucosal cell populations [8, 12]. An improved method of sampling the nasal mucosa would lead to a greater understanding of the cellular components of the immune response in the nasopharynx. Cell collection using nasal curettes has previously been used to collect epithelial cells for culture, as well as for gene expression analysis [13, 14]. Nasal brushes have also been used to collect samples to investigate epithelial cell phenotype [15]. Here we use for the first time cell collection with nasal curettes to analyse the composition and activation state of immune cells using flow cytometry. A NW is also typically performed to measure cytokine and other soluble immune mediators. An absorptive matrix to collect nasal fluid (nasosorption) has been used in neonates, and has the potential to be better tolerated and more widely applicable [16]. This technique has recently been used to investigate nasal responses to grass pollen, LPS and rhinovirus [17–19]. Here, we aimed to compare cytokine detection between nasal wash and nasosorption techniques.

Here we describe the novel use of two non-invasive nasal microsampling techniques. We present data on the reproducibility, utility and tolerability of these techniques in measuring immunological responses within the nasal mucosa.

## Methods

### Recruitment of volunteers and ethical statements

We recruited healthy non-smoking adults aged between 18–60 years of age. Volunteers gave written informed consent. Inclusion criteria were: capacity to give informed consent, aged 18–50 years and speak fluent English. Exclusion criteria were: current involvement in another study unless observational or in follow-up phase (non-interventional), influenza vaccination in the last 2 years, clinically diagnosed with influenza in the last 2 years, egg allergy, previous significant adverse reaction to any vaccination/immunisation, close contact with at risk individuals (children under 5 years, immunosuppressed adults, elderly, chronic ill health), current regular smoker, >10 pack years smoking history, asthma or respiratory disease, pregnant, women of child-bearing potential without effective birth control in place, allergic to penicillin/amoxicillin/ gentamicin, on medication that may affect the immune system in any way e.g. steroids, steroid nasal spray, regularly taking acetylsalicylic acid (aspirin), been involved in a clinical trial involving experimental pneumococcal carriage over the last 3 years, current acute severe febrile illness, taking long term antibiotics. Volunteers were screened for *S*. *pneumoniae* carriage and only carriage-negative volunteers were analysed. In addition, volunteers with nasal and general symptoms were excluded from the analysis. Ethical approval was given by NHS Research and Ethics Committee (REC)/Liverpool School of Tropical Medicine (LSTM) REC, reference numbers: 15/NW/0146 and 14/NW/1460 and Human Tissue Authority licensing number 12548. The individuals in this manuscript have given written informed consent (as outlined in PLOS consent form) to publish these case details

### Nasal sampling procedures

All participants underwent nasal wash procedures as described previously using 20mL of sterile saline [11]. A selection of participants underwent nasal curettage and nasosorption. Nasal curettage uses a small probe to collect cells from the nasal mucosa (S1 Fig). The nasal inferior turbinates were visualised with a light with the participant being seated with the head tilted posteriorly. The curette (ASL Rhino-Pro^©^, Arlington Scientific) was used to scrape a small collection of cells from the nasal mucosa (S1 Video). Two scrapes per nostril were taken and placed in a 15mL Falcon tube placed on ice containing phosphate-buffered saline (PBS) + 0.5% heat-inactivated fetal bovine serum (FBS) and 2.5 mM ethylenediaminetetraacetic acid (EDTA) (PBS++, all ThermoFisher). For nasosorption collection, an adsorptive matrix strip (Nasosorption™, Hunt Developments) was inserted into the nostril and placed against the nasal lining for 2 minutes and then placed in its transport tube (S2 Fig).

### Flow cytometry analysis

Cells were dislodged from the curette by repeated pipetting with PBS+. Cells were spun down (440 x g for 5 minutes) and resuspended in PBS++ containing LIVE/DEAD® Fixable Aqua Dead Cell Stain (ThermoFisher). After 15 minute incubation on ice, an antibody cocktail containing Epcam-PE, HLADR-PECy7, CD16-APC, CD66b-FITC (all Biolegend), CD3-APCCy7, CD14-PercpCy5.5 (BD Biosciences) and CD45-PACOrange (ThermoFisher) was added to the cells. Following a further 15 minute incubation on ice, cells were filtered over a 70 μm filter (ThermoFisher). Cells were spun down (440 x g for 5 minutes), resuspended in PBS++ and acquired on a flow cytometer (LSRII, BD). Samples with less than five hundred events (15% of all measured) were excluded from further analysis. Nasal washes were similarly processed excluding the dislodging step. To 100 μL heparinized blood, the viability dye and antibodies were directly added sequentially. After the final incubation step, BD FACS lysing solution was used to remove red blood cells according to manufacturer’s instruction. Flow cytometry data was analysed using Flowjo V. 10 (Treestar).

### Cytokine detection

Nasal lining fluid was extracted from nasosorption strips by centrifugation (1880 x g for 10 minutes) and frozen until use at -80C. Supernatant from nasal wash was collected by centrifugation at 3000 rpm for 3 minutes and was stored at -80C until use. Interferon gamma-induced protein 10 (IP10), interleukin 8 (IL-8) and monocyte-chemoattractant protein 1 (MCP1) were measured by enzyme-linked immunosorbent assay (ELISA) (BD OPTEIA) according to manufacturer’s instructions. The human magnetic 30-plex cytokine kit (ThermoFisher) was used to detect thirty cytokines simultaneously on a LX200 with xPonent3.1 software (Luminex) following manufacturer’s instructions.

### Scoring tolerability of nasal sampling procedures

Following nasal wash, nasal curettage and nasosorption, participants rated by 5-point modified Likert scale how ‘painful’ and how ‘uncomfortable’ each procedure was, and how much it made their ‘eyes water’. Thirty-nine participants also completed a symptoms log for 7 days documenting both local and general symptoms with severity ratings from 1–7.

### Multi-dimensional scaling and heat map generation

Multi-dimensional scaling and heat map representations were generated using R. Flow cytometry data (epithelial cell yield, immune composition and activation) was log-transformed and a distance-matrix was calculated. The Kruskal stress was calculated using the ‘MASS’ package. Heat maps of log-transformed cytokine data were generated using the ‘gplots’ package

### Statistical analysis

Non-parametric two-tailed tests were used throughout using Prism 5 (Graphpad). If two groups were compared, a Mann-Whitney test was used. If multiple groups were compared, a Kruskal-Wallis test was used, followed by a Dunn’s post-test. A Spearman test was used to measure correlations between two continuous variables. Analysis of similarity (ANOSIM) testing was performed using the ‘vegan’ package in R.

## Results

### Nasal curettage yields robust and reproducible data

**Fig 1:**
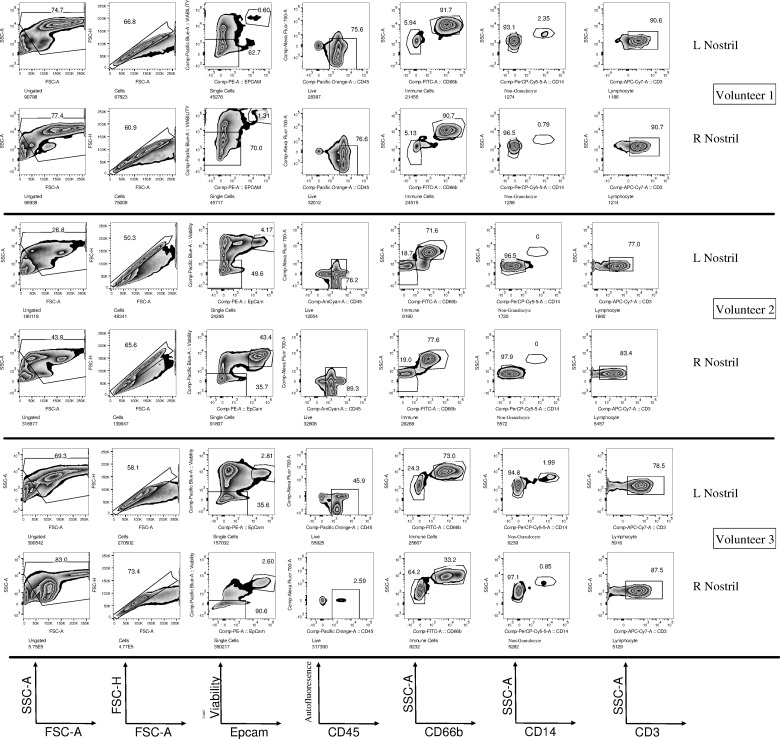
Repeatability of nasal curettage sampling. Nasal cells were collected from the left (L) and right (R) nostril of three volunteers, processed independently and their composition was assessed by flow cytometry. After excluding debris and doublets, epithelial cells were identified by Epcam expression. A viability dye and CD45 were used to identify live immune cells. Among those cells, side scatter, CD66b, CD14 and CD3 were used to identify granulocytes, monocytes and T cells respectively.

We collected nasal cells from the inferior turbinate using curettes (Supplementary Video 1). In total 240 samples were collected from 139 healthy individuals to investigate their use for studying immune responses at the mucosal level. To verify the repeatability of nasal curettage, we initially collected samples from the left and right nostril of three healthy volunteers and performed flow cytometry to identify cellular composition (Fig 1). Samples from both nostrils were processed independently and frequencies of granulocytes, monocytes and T cells were compared for each of the three volunteers. Cellular samples collected from the two nostrils were similar for each of the three volunteers, compared to samples collected from the other two volunteers. This demonstrates the repeatability of nasal curettage as well as the presence of inter-individual variation in immune cells in the nose.

**Fig 2:**
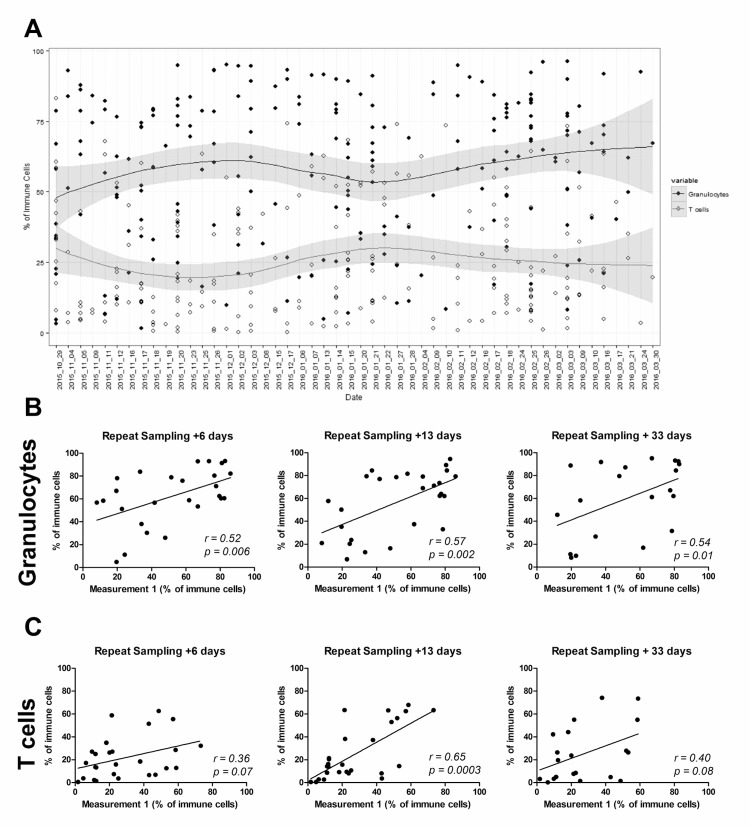
Nasal curettage yields reproducible and consistent results over time. (A) The percentage of granulocytes (closed circles) and T cells (open circles) in 218 nasal cell samples collected over a five month period (n = 117 volunteers, sampled up to five times). Individual samples and loess curves are depicted for both populations. (B, C) The correlation for individuals in four repeated measurements over a 33-day period for (B) granulocytes and (C) T cells.

To verify that nasal curette sampling yields stable data, cells were collected from healthy volunteers (n = 117) over a five month period (Fig 2). The percentage of granulocytes and T cells among immune cells was stable during this period (Fig 2A). Moreover, for a subset of volunteers, up to four nasal samples were collected during a thirty-three day period. The levels of both granulocytes and T cells correlated on an intra-individual level between repeated sampling (Fig 2B and 2C). This demonstrates that despite variation between individuals, the immunological profile in the nose is stable in the absence of disease or immune intervention such as vaccination.

### Nasal curettage and nasal wash yield different cell populations

**Fig 3:**
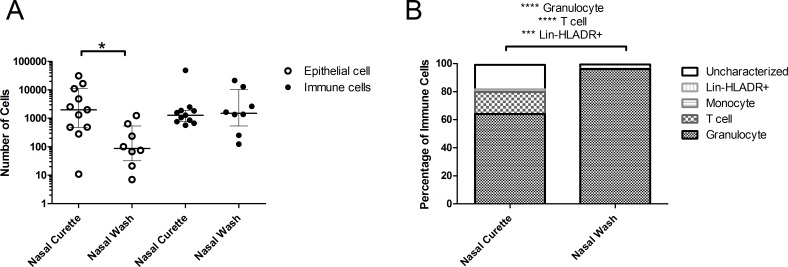
Comparison of samples collected by nasal wash and nasal curette. (A) Epithelial (open circles) and immune (closed circles) and cell yields were compared between nasal wash pellets and nasal curette samples. Individuals samples and median and interquartile range are shown. (B) Median proportions of granulocytes, T cells, monocytes, lineage^-^ HLA-DR^+^ and uncharacterized cells among immune cells in nasal curette (n = 139 individuals) and nasal wash (n = 8) samples. *p < 0.05, ***p < 0.001, ****p < 0.0001 Mann-Whitney test.

We then compared the yield and composition of nasal cells collected using curettes to those collected using a NW, the currently most used method for collecting nasopharyngeal cells (Fig 3A and 3B). Nasal curettage yielded a median of 4367 (interquartile range (IQR): 1511–10348) immune cells and 1407 (IQR: 570–3194) epithelial cells, respectively. The number of immune cells obtained was similar between NW and nasal curette. In contrast, there were 22.7 fold increased numbers of epithelial cells collected by nasal curette (Fig 3A, p < 0.05). Fig 3B shows the composition of the collected immune cell. NW immune cells consisted almost exclusively of granulocytes (median 96%, IQR: 93–97%). In contrast, nasal curette samples contained predominantly granulocytes (median 64%, IQR: 39–79%, p < 0.0001 compared to NW), but also consisted of a larger fraction of T cells than NW (median 16%, IQR: 9–38%, p < 0.0001 compared to NW). A median of 2591 (IQR: 691–7666) granulocytes and 633 (IQR: 210–1740) T cells were acquired per sample. Nasal curette samples also contained more lineage^-^ human leukocyte antigen-antigen D related (HLA-DR)^+^ cells, which are likely to consist of B cells and dendritic cells (median 1.7%, IQR 0.9–3.2%, p < 0.001 compared to NW). Of all immune cells collected by curettage and NW, 81.7% and 96.4% could be characterized, respectively.

### Differences in proportions and activation state of immune cells in blood and nasal cells

**Fig 4:**
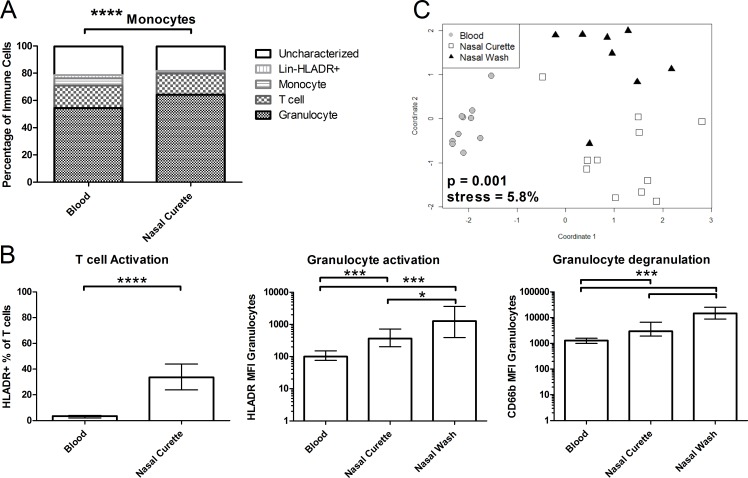
Comparison of samples from nasal mucosa and blood. (A) Median proportions of granulocytes, T cells, monocytes, lineage^-^ HLA-DR^+^ and uncharacterized cells among immune cells in blood (n = 10) and nasal curette (n = 139). **** p < 0.0001 Mann-Whitney test. (B) The percentage of HLA-DR^+^ T cells in blood and nasal curette samples and mean fluorescent intensity (MFI) of HLA-DR and CD66b on granulocytes was measured for blood, nasal curette and nasal wash (n = 8) samples. Median and interquartile range are shown. *p < 0.05, ***p < 0.001 Kruskal-Wallis, followed by Dunn’s Multiple Comparison Test. (C) Multi-dimensional scaling analysis shows the clustering of samples from blood (grey circles), nasal curette (open squares, 11 randomly selected) and nasal wash (black triangles). The epithelial cell yield, activation state of granulocytes and composition of the immune cells were taken into account. Kruskal stress = 5.8% and Analysis of Similarity ANOSIM p-value = 0.001.

Next, we compared immune populations from nasal samples with those found in blood (Fig 4A). Levels of neutrophils, T cells and Lineage^-^HLA-DR^+^ were similar between nasal curette samples and blood. In contrast, levels of monocytes/macrophages were decreased in nasal curette samples compared to blood (0.3% and 5.4%, respectively, p < 0.0001). Immune cells from nasal mucosa differed from blood cells in their activation state (Fig 4B). Levels of HLA-DR^+^ T cells were significantly increased in nasal curette samples compared to blood (p < 0.0001). Similarly, levels of HLA-DR were significantly increased on granulocytes in nasal curette samples and NW samples compared to blood (p < 0.001). Granulocytes from nasal curette samples and NW samples also displayed high CD66b expression relative to blood (p < 0.001), a marker for degranulation. Granulocyte activation and degranulation was even further increased in NW compared to nasal curette samples (p < 0.05 and p < 0.001, respectively). When taking epithelial cell yield, immune cell composition and activation state into account, nasal curette samples clustered separately from blood and NW (Fig 4C).

### Macroscopic red blood cell contamination does not affect immune cells recovered using nasal curettage

**Fig 5:**
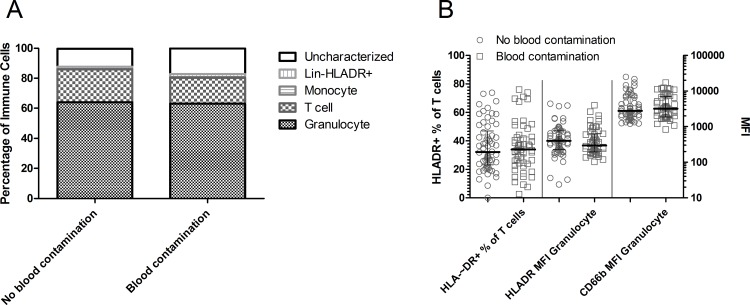
Comparison of nasal curettes with visible red blood cell contamination (n = 55) or not (n = 62). (A) Median proportions of granulocytes, T cells, monocytes, lineage^-^ HLA-DR^+^ and uncharacterized cells among immune cells. (B) The percentage of HLA-DR^+^ T cells (left axis) and mean fluorescent intensity (MFI) of HLA-DR and CD66b on granulocytes (right axis) of nasal curette samples visibly contaminated with erythrocytes (open squares) or not (open circles). Individuals samples and median and interquartile range are shown.

Red blood cells were visible macroscopically in 44% of collected nasal curette samples (52 of 117 assessed). Blood leukocytes did not contaminate the recovered immune cells in these samples, as levels of monocytes among immune cells were not increased in the presence of red blood cells (Fig 5A). Moreover, T cell activation and neutrophil activation and degranulation were not decreased when red blood cells were present (Fig 5B).

### Cytokine detection from nasal lining fluid using nasosorption devices

**Fig 6:**
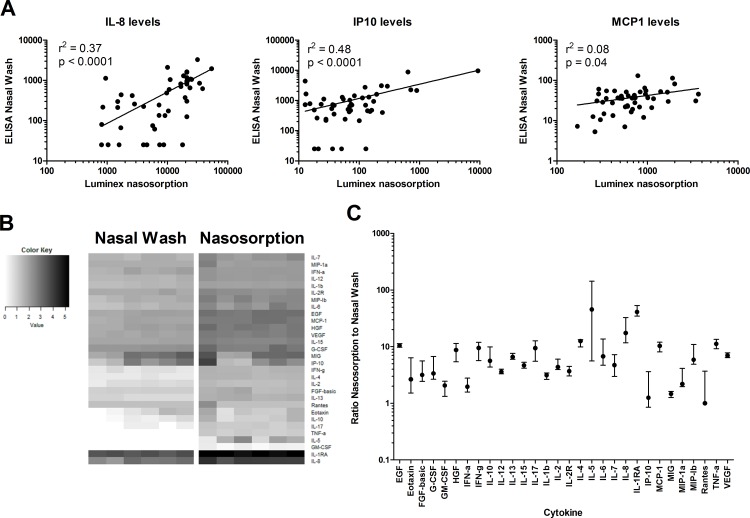
Comparison of cytokine levels in samples collected by nasal wash and using nasosorption devices. (A) Levels of IL-8, IP-10 and MCP1 were measured by Luminex in nasosorption devices and by ELISA from paired nasal washes (n = 41). The r^2^ and p indicate goodness-of-fit and p-value calculated by linear regression analysis (B) A heat map depicts log-transformed cytokine concentrations, with white and black indicating low and high levels, respectively. Each of the columns corresponds to one sample (n = 6 nasal wash and nasosorption) and each of the rows to one cytokine. A legend assigning color gradient to log-transformed cytokine levels is displayed. (C) The ratio of cytokine concentrations measured in paired nasosorption and nasal wash (median and IQR are shown, n = 6).

To investigate cytokines in the nose, we used nasosorption devices to collect nasal lining fluid. The median volume of nasal lining fluid returned using this technique was 42.5μL (IQR: 29.25–71.25 μL, n = 41). We measured the levels of thirty cytokines in these samples by multiplex ELISA. To investigate whether concentrations of cytokines collected locally by nasosorption can be used to detect intra-individual differences, we measured levels of three cytokines by ELISA in paired NW samples as a control (Fig 6A). Levels of IL-8 (r^2^ = 0.37, p < 0.0001), IP10 (r^2^ = 0.48, p < 0.0001) and MCP1; r^2^ = 0.08, p = 0.04) correlated across the two sample types and detection techniques.

We then compared cytokine levels in nasal lining fluid and NW supernatant for thirty cytokines by Luminex (Fig 6B). Levels of different cytokines varied considerably, with median levels of IL1 receptor antagonist (IL-1RA) at 212,000 pg/mL and granulocyte macrophage colony-stimulating factor (GM-CSF) at 2 pg/mL in nasal lining fluid. Relative cytokine abundancy correlated well between nasosorption and NW such that cytokines that were abundant in nasosorption were also highly present in NW. Of interest, T cell cytokines (IL-10, IL-17, Interferon gamma (IFNγ), Tumour necrosis factor alpha (TNFα), IL-4, IL-5, IL-2) were only present at low levels. This correlates well with the absence of T cells in the lumen (Fig 3B). Growth factors as epidermal growth factor (EGF), hepatocyte growth factor (HGF) and vascular endothelial growth factor (VEGF) were expressed at moderately high levels, reflecting the homeostatic nature of mucosal surfaces.

Levels of cytokines were higher in nasosorption compared to NW (median 4.7x, IQR: 3.1–8.0x; Fig 6C). However, some cytokines had a ratio between nasosorption and NW that differed substantially from this: IL-1RA and IL-5 were respectively 51.5x and 45.2x higher in nasosorption than in NW. In contrast, Monokine induced by IFNγ (MIG), Regulated on activation, normal T-cell expressed and secreted (Rantes) and IP10 were found at similar levels in nasosorption and in NW (ratio of 1.5x, 1.0x and 0.9x, respectively).

While all cytokines were within the limit of detection of the assay (0.5 pg/mL) in the nasal lining fluid, several cytokines could not be detected in one or more NW samples. Five cytokines (GM-CSF, TNFα, Rantes, IL-5 and Macrophage inflammatory protein (MIP)-1a) were not detectable in the NW of any of the six volunteers. One cytokine (IL-17) could not be detected in 3/6 samples. Two cytokines, IL-2 Receptor (IL-2R) and G-CSF, were below the detection limit for 2/6 samples and an additional 9 cytokines were undetectable in 1/6 NW samples.

### Methods of nasal microsampling are well tolerated by volunteers and do not lead to symptoms

**Fig 7:**
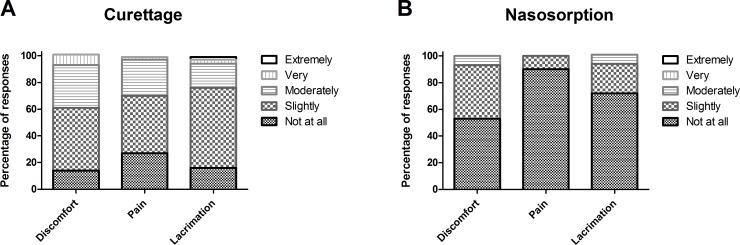
Tolerability of novel nasal sampling methods. The percentage of volunteers rating (A) nasal curettage and (B) nasosorption on discomfort, pain and lacrimation.

Using the 5-point modified Likert scale, twenty participants gave ratings for nasal curettage (eighty-eight ratings) and nasosorption (sixty ratings) with regards to pain, discomfort and lacrimation. For nasal curettage and nasosorption, the proportion of responses that reported any degree of pain (any score > 1) were 73% and 10%, respectively (Fig 7). For nasal curettage and nasosorption, the proportion of responses that reported any degree of discomfort (score > 1) were 86% and 47%, respectively Fig 7). For nasal curettage and nasosorption, the proportion of responses that reported any degree of lacrimation (score > 1) were, 84% and 28%, respectively (Fig 7). For nasosorption the maximum rating was moderately for levels of discomfort, pain or causing lacrimation. A small proportion of responses rated nasal curettage as very painful (2%), uncomfortable (8%) or causing lacrimation (6%).

Finally, we assessed whether these sampling techniques led to increased general and nasal symptoms over a longer period in thirty-nine healthy volunteers (S1 Table). All participants had nasal wash procedures and twenty of those participants had nasal curettage and nasosorption to investigate whether these additional sampling methods affect nasal symptoms. Daily symptom logs for nasal and general symptoms were completed by all volunteers. Age and sex distribution was similar in each group. The median ratings for overall nasal symptoms were 1 (range 1–4) and 1 (range 1–5) in the group with and without additional nasal sampling, respectively. In the symptoms log, a score of 1 represented ‘none to occasional symptoms’ and 5 represented ‘moderately bothersome’. Comparison of area under the curve between the two groups showed no significant difference in either overall nasal symptoms or general symptoms. In addition, we compared nasal and general symptoms between participants with and without additional nasal sampling on day 1 (before additional sampling) and day 3 (after additional sampling). There were no significant differences between nasal or general symptoms with or without additional nasal sampling at these time points.

## Discussion

We have shown that non-invasive sampling of the nasal mucosa is well tolerated and reproducible, yielding immune cells that are stable over time. In particular, our results demonstrate the reliability of nasal curettage as a method for studying the nasal immune response. The ability to measure cellular phenotype during infection could shed light on the mechanisms that are involved in pathogen clearance. This technique could also be used in vaccine testing to define cellular correlates of protection at a mucosal level [20]. Moreover, the use of these sampling techniques will facilitate the investigation of cellular responses in asthma, which includes an important immunological component of the upper respiratory tract [21].

Immune cells from the nose differed from those in the blood in composition and activation state. Both T cells and granulocytes collected from the nose displayed an increased activation profile compared to blood leukocytes, consistent with earlier reports [9]. Moreover, luminal granulocytes had an increased activation profile compared to those found using nasal curettes. As neutrophil migration through the epithelial layer depends on interaction with molecules as Intercellular adhesion molecule 1 (ICAM-1) [22], it is possible that these granulocytes become activated by the transmigration event itself.

The only difference in proportion of immune cells found between blood and nasal mucosa was the decreased presence of monocytes in nasal samples. It has been demonstrated that intestinal macrophages lack CD-14 expression [23]. Staining with additional macrophage markers (CD163 and CD206) confirmed the low levels of monocytes/macrophages in nasal mucosa (S3 Fig). Similar to our findings, monocytes were described to be present only at low levels in nasal aspirates of healthy children [24]. In the presence of influenza A infection however, these cells were recruited to the nose [24]. It will be useful to compare nasal cell profiles from healthy and diseased individuals using this new sampling technique.

Differences in immune cells were also present between NW and nasal curette samples as NW yielded almost exclusively granulocytes. The lack of T cells in NW reflects earlier findings in other body compartments: neutrophils readily enter the lumen in the gut, while T cells are mostly associated with the epithelial layer [8, 12]. The nasal curette sampling method allows the collection of such sub-epithelial and intra-epithelial cell populations, while NW is limited to sampling luminal populations. However, Natural killer (NK) cells and epithelial cells were found at high levels in nasal washes in a study by Horvath *et al* [25]. As this study collected NW through 40 sprays of 100uL of saline followed by forceful expulsion rather than the more gentle NW approach we employed [11], it is possible that different collection NW techniques yield different cell populations. Our flow cytometry panel did not include markers that could assess NK cell frequency in samples collected using NW or curettes. Increased collection of epithelial cells with nasal curettage compared to NW is likely due to the technique itself (S1 Video) rather than a difference in sample sites.

Although blood and nasal immune cells displayed different profiles, a visible presence of red blood cells in nasal samples did not change the phenotype of the collected nasal cells. As the level of leukocytes in blood is relatively low it is not surprising that the presence of red blood cells at macroscopically visible levels does not indicate a substantial blood leukocyte contamination [26].

Of all immune cells, eighty percent could be identified in both blood and nasal curette samples. The remaining twenty percent like consists of various types of cells that could not be analyzed using this flow cytometry panel, such as innate lymphocytes and basophils. Increasing the number of markers will allow the further study of this fraction, which could potentially identify other differences in immune composition between blood and nose. As cell yields are low, the capacity to study rare cell populations or perform functional assays using such collected cells is limited however.

The second nasal sampling technique that we assessed was the use of nasosorption devices to collect nasal lining fluid. Importantly, the nasal lining fluid contained cytokines in concentrations that were increased compared to NW, allowing for the investigation of several cytokines that were undetectable in NW supernatant. A correlation was seen between cytokine levels that were detected in both nasal lining fluid and those from NW. However, the ratio between cytokine concentrations in nasal lining fluid and NW was not similar for all cytokines assessed. One potential explanation is differences in levels could exist for some cytokines in different nasopharyngeal compartments. As nasal washes sample the entire nasopharynx in contrast to a localized sample coming from the nasosorption strip this might lead to different returned cytokine levels. Another possible explanation is that differences exist between cytokines in their propensity to bind to the nasosorption paper. NW is currently the most commonly used method to obtain samples from the nasopharynx. Despite this, it has limited application in multiple clinical scenarios. Here we have demonstrated that nasosorption has greater sensitivity than the traditional wash and is extremely well tolerated (Fig 7). A potential problem with sample collection using nasosorption devices can be poor return volume, in particular if volunteers are dehydrated.

In conclusion, non-invasive mucosal sampling yields nasal cells and nasal lining fluid that can be used to study both cellular and soluble immune responses at the mucosal surface. Such sampling is well-tolerated and does not lead to a change in nasal symptoms. These techniques can be easily implemented and provide researchers with an effective tool to study immunological parameters in the upper respiratory tract.

## Supporting Information

S1 Fig Image of nasal curette (ASL Rhino-Pro^©^, Arlington Scientific) before insertion into nostril. (TIF)

S2 Fig Image of adsorptive matrix strip (Nasosorption™, Hunt Developments) before insertion into nostril. (TIF)

S3 Fig Flow cytometry data for one volunteer showing with absence of CD163 and CD206 staining in the non-granulocyte immune gate. Some CD14 positive events can be seen for this volunteer indicating the presence of monocytes in the sample.
(TIF)

S1 Table Demographics and symptoms of the groups with and without additional nasal sampling. (DOCX)

S1 Video Supplementary video demonstrating correct nasal curette sampling technique. The curette is placed on the inferior turbinate and then dragged forward to collect cells from the nasal lining.
(MP4)
