# Supplementary figures and images for: Novel Analysis of Immune Cells from Nasal Microbiopsy Demonstrates Reliable, Reproducible Data for Immune Populations, and Superior Cytokine Detection Compared to Nasal Wash
Source: PLoS One. 2017 Jan 20;12(1):e0169805. doi: 10.1371/journal.pone.0169805 (PMC5249128; doi:10.1371/journal.pone.0169805)

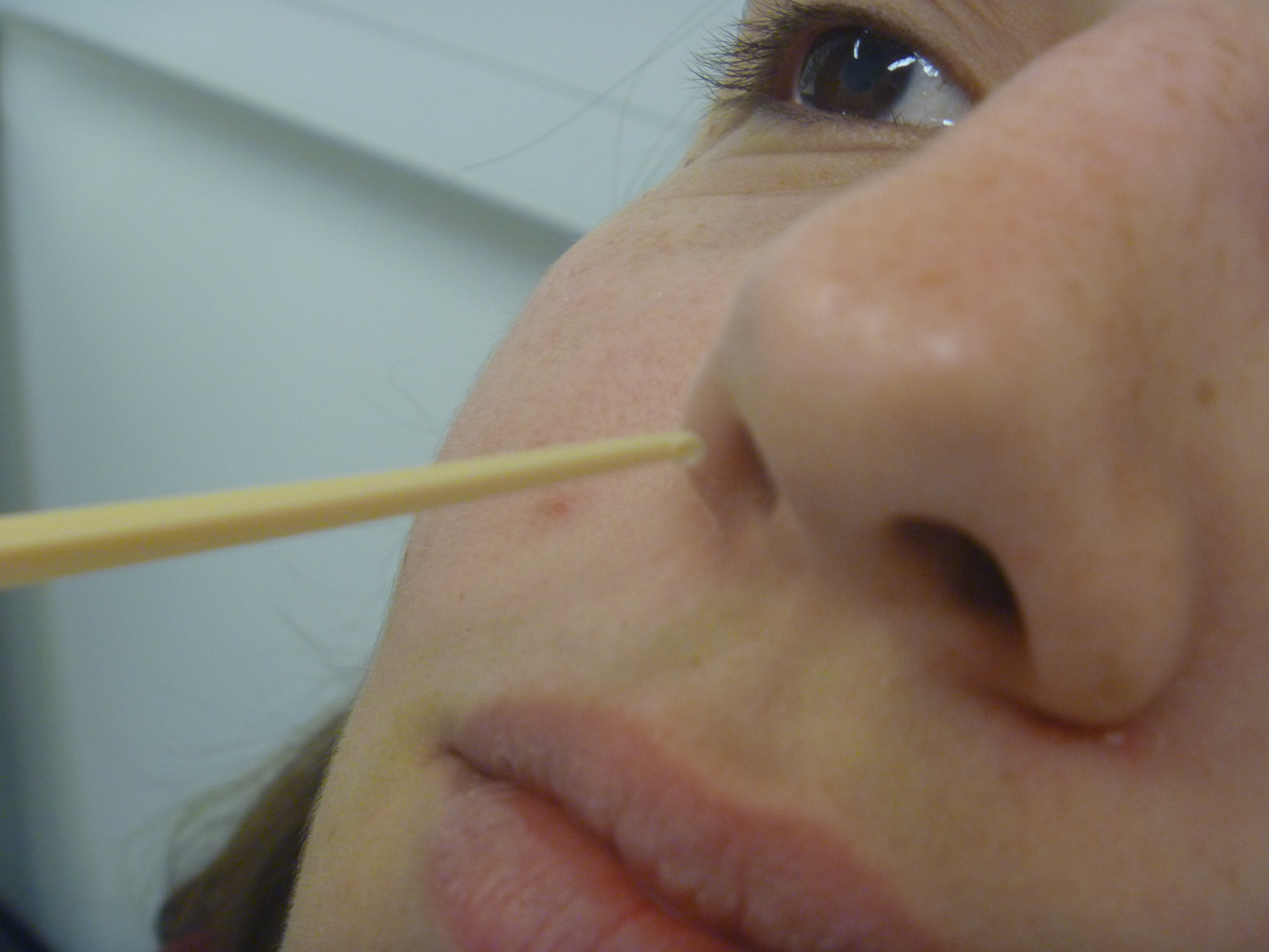

Supplement: S1 Fig — (TIF) [file pone.0169805.s001.tif]

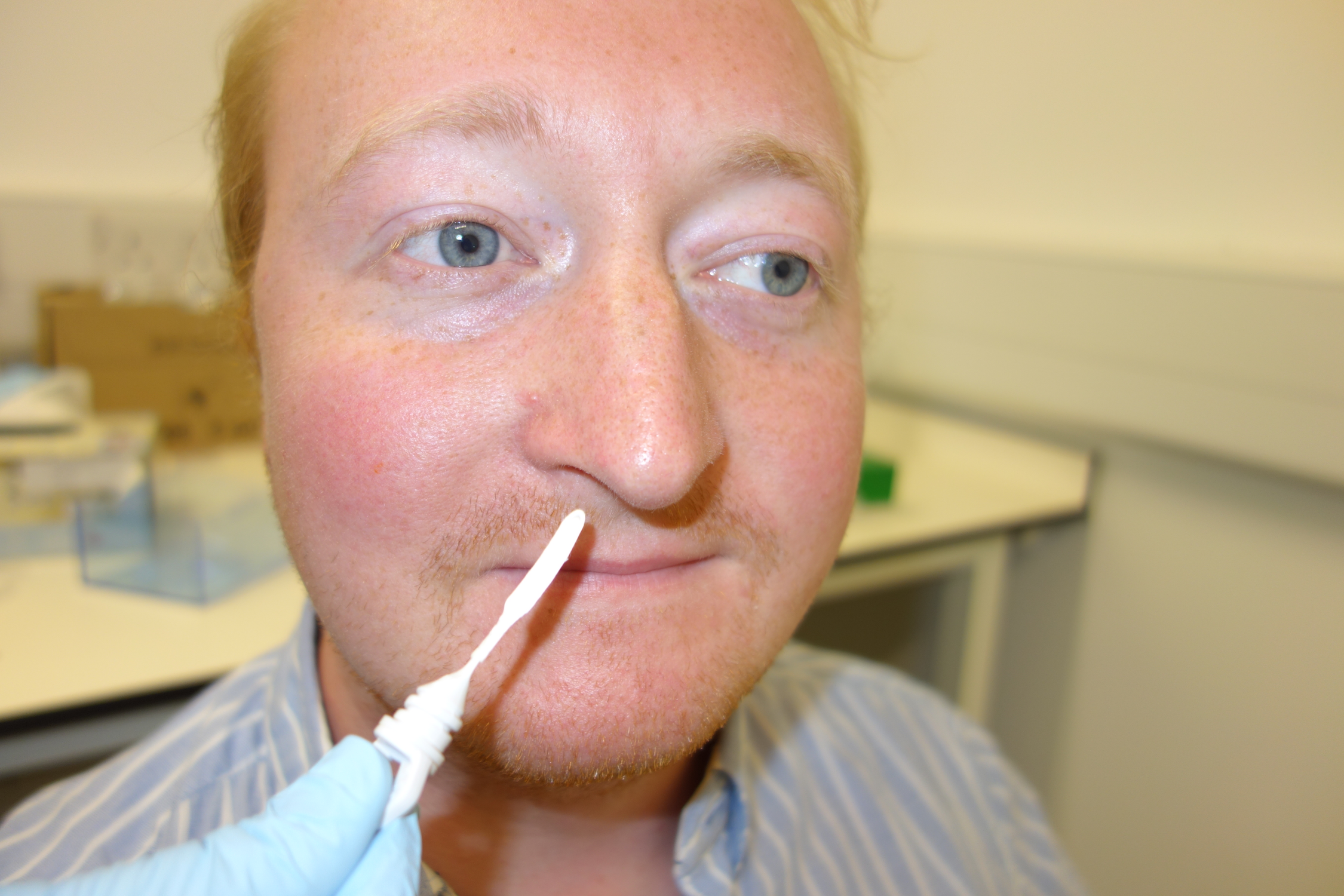

Supplement: S2 Fig — (TIF) [file pone.0169805.s002.tif]

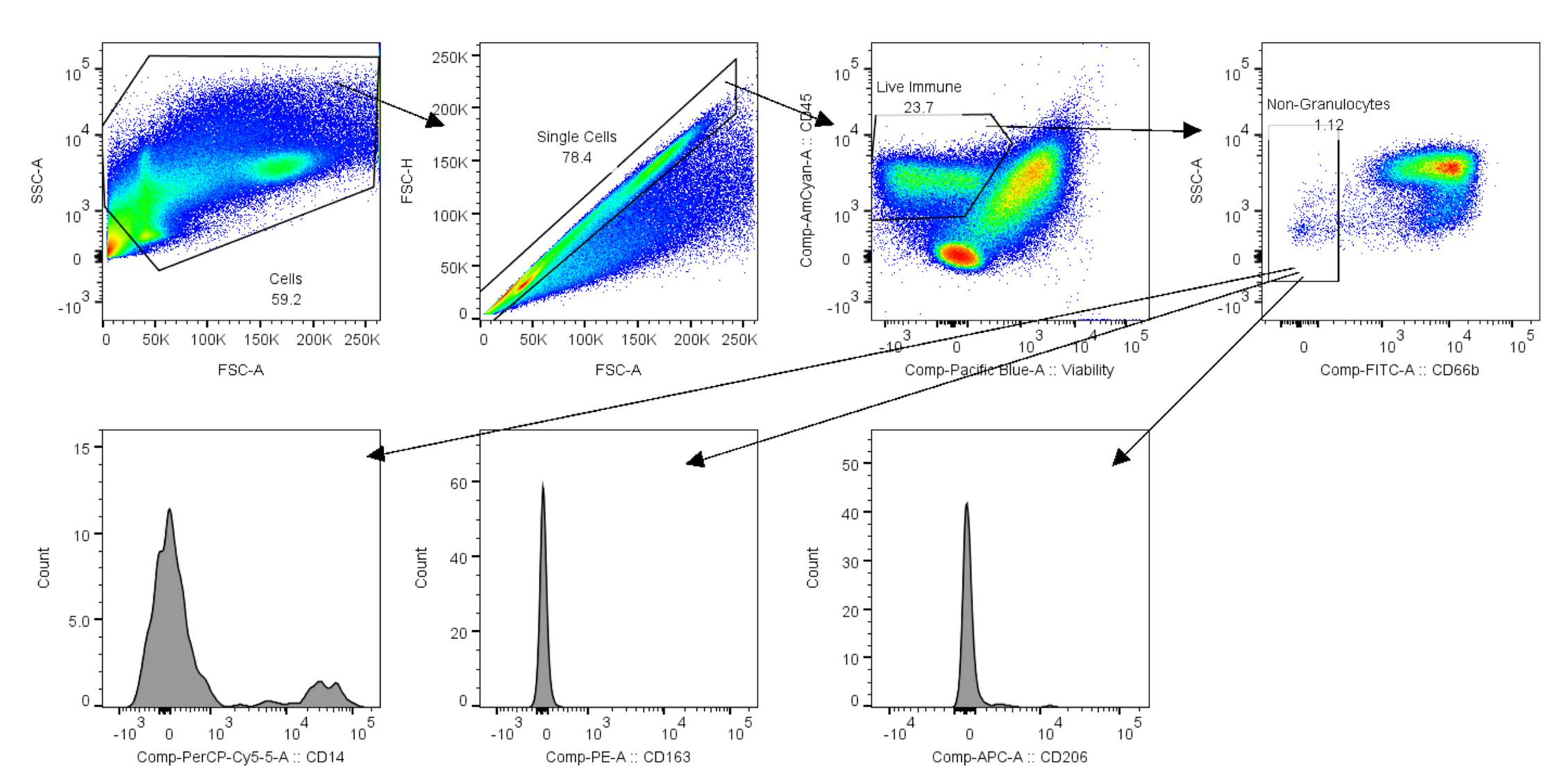

Supplement: S3 Fig — Some CD14 positive events can be seen for this volunteer indicating the presence of monocytes in the sample. (TIF) [file pone.0169805.s003.tif]
