# Supplementary material for: Novel Analysis of Immune Cells from Nasal Microbiopsy Demonstrates Reliable, Reproducible Data for Immune Populations, and Superior Cytokine Detection Compared to Nasal Wash
Source: PLoS One. 2017 Jan 20;12(1):e0169805. doi: 10.1371/journal.pone.0169805 (PMC5249128; doi:10.1371/journal.pone.0169805)
Supplement: S1 Table — (DOCX) [file pone.0169805.s004.docx]

|  | With additional nasal sampling | Without additional nasal sampling |
| --- | --- | --- |
| Number of participants | 20 | 19 |
| Age Range | 19-46 | 20-32 |
| Male (%) | 7/20 (35) | 7/19 (45) |
| Nasal Symptoms median (Range 1-7) | 1 (1-4) | 1 (1-5) |

**S5 Table. Demographics and symptoms of the groups with and without additional nasal sampling**
